# Identification of the type II cytochrome *c* maturation pathway in anammox bacteria by comparative genomics

**DOI:** 10.1186/1471-2180-13-265

**Published:** 2013-11-23

**Authors:** Christina Ferousi, Daan R Speth, Joachim Reimann, Huub JM Op den Camp, James WA Allen, Jan TM Keltjens, Mike SM Jetten

**Affiliations:** 1 Department of Microbiology, Institute for Water and Wetland Research, Radboud University Nijmegen, Heyendaalseweg 135, 6525, AJ, Nijmegen, the Netherlands; 2 Department of Biochemistry, University of Oxford, South Parks Road, OX13QU Oxford, UK

**Keywords:** Cytochrome *c* biogenesis, Anaerobic ammonium oxidizing bacteria, ccs, CcsA, CcsB, CcsX, DsbD, CcdA

## Abstract

**Background:**

Anaerobic ammonium oxidizing (anammox) bacteria may contribute up to 50% to the global nitrogen production, and are, thus, key players of the global nitrogen cycle. The molecular mechanism of anammox was recently elucidated and is suggested to proceed through a branched respiratory chain. This chain involves an exceptionally high number of *c*-type cytochrome proteins which are localized within the anammoxosome, a unique subcellular organelle. During transport into the organelle the *c*-type cytochrome apoproteins need to be post-translationally processed so that heme groups become covalently attached to them, resulting in mature *c*-type cytochrome proteins.

**Results:**

In this study, a comparative genome analysis was performed to identify the cytochrome *c* maturation system employed by anammox bacteria. Our results show that all available anammox genome assemblies contain a complete type II cytochrome *c* maturation system.

**Conclusions:**

Our working model suggests that this machinery is localized at the anammoxosome membrane which is assumed to be the locus of anammox catabolism. These findings will stimulate further studies in dissecting the molecular and cellular basis of cytochrome *c* biogenesis in anammox bacteria.

## Background

**Figure 1:**
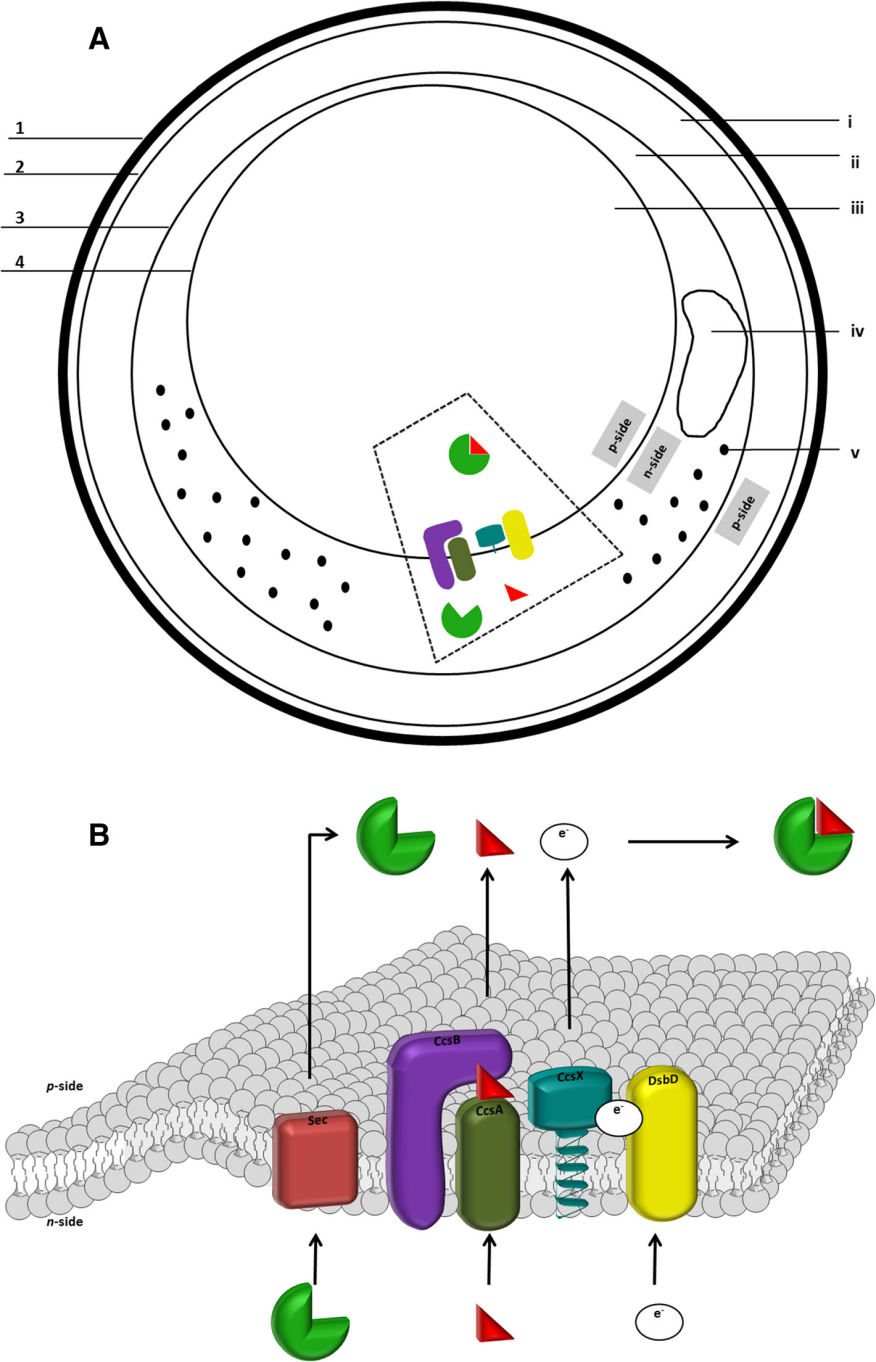
**Maturation System II of *c*-type cytochrome proteins in anammox bacteria. A**: Schematic drawing of the anammox cell and the maturation system machinery depicted on it. The dotted trapezoid is zoomed-in in Figure 2B. 1: cell wall; 2: cytoplasmic membrane; 3: intracytoplasmic membrane; 4: anammoxosome membrane; i: paryphoplasm; ii: riboplasm; iii: anammoxosome; iv: nucleoid; v: ribosome. **B**: 3D illustration of cytochrome *c* maturation System II localized within the anammoxosome membrane. Apocytochrome *c* is translocated to the *p*-side of the membrane via the Sec pathway. CcsA-CcsB complex, forming the heme channel entry, is tethered within the anammoxosome membrane. Heme is, thus, translocated within the anammoxosome. Concurrently, reducing equivalents from the *n*-side of the cell are fed to a disulfide bond cascade that proceeds from DsbD to CcsX. The latter, being a dedicated thiol-disulfide oxidoreductase, reduces the cysteine residues of apocytochrome *c*, and eventually spontaneous ligation for the thioether linkages formation between the apoprotein and its cofactor takes place. Green pie depicts apocytochrome *c*; red triangle depicts heme molecule.

One of the most recent additions to the microbial nitrogen cycle is the anaerobic oxidation of ammonium (anammox), which utilizes nitrite as the electron acceptor and forms dinitrogen gas under anaerobic conditions. Anammox bacteria possess intracellular membrane systems, leading to a remarkable cell compartmentalization [1]. Two membranes on the inner side of the protein-rich cell wall form a ribosome-free peripheral compartment, the paryphoplasm [2]. A third and innermost bilayer membrane exhibits a highly curved configuration and further separates the cytoplasm into two distinct regions, namely the riboplasm and the anammoxosome (Figure 1A). Detailed electron microscopic and labeling studies strongly support the hypothesis of the anammoxosome being a separate organelle, where the central anammox catabolism resides [1,3,4]. The annotation of more than 200 genes involved in catabolism and respiration in the genome of the anammox bacterium *Kuenenia stuttgartiensis*, together with the abundance of 61 genes encoding *c*-type cytochrome proteins, reflects the complexity of the anammox metabolism and implies the presence of a branched and versatile respiratory chain [5]. This complexity is further confirmed by the genome assemblies of two more anammox species that were recently reported (*Scalindua profunda*[6]; strain KSU-1 [7]). Although *c*-type cytochrome proteins seem to play a key role in the unique anammox metabolism, the maturation pathway of functional *c*-type cytochrome holoforms has not been explored. Cytochrome *c* maturation describes the post-translational process by which *b*-type hemes (Fe-protoporphyrin IX) are covalently attached to the apoproteins resulting in functional *c*-type cytochromes. After synthesis, apocytochrome *c* and heme molecules are independently translocated across the energy-transducing membrane into the bacterial periplasm, the mitochondrial intermembrane space or the thylakoid lumen. Ferric iron of heme(s) and cysteine residues of apocytochrome *c* are reduced and subsequent thioether linkage formation occurs between the heme vinyl groups and the CX_2-4_CH sulfhydryls of apocytochrome *c*, leading to the functional holoform [8]. Three distinct cytochrome *c* maturation pathways (Systems I, II and III) have been described, each comprising system-specific assembly protein complexes; these biogenesis systems occur in a wide variety of organisms with a complex and unpredictable phylogenetic distribution [9].

Considering the remarkable anammox cell plan together with the high abundance of cytochrome *c*-type proteins, determination of the cytochrome *c* maturation system that these bacteria employ is of particular importance. In this study, comparative computational methods were applied to determine the maturation pathway regulating the assembly of functional *c*-type cytochrome holoforms in four genera of anammox bacteria, using key protein constituents of maturation Systems I-III as biomarkers. Our analysis showed that all anammox genome assemblies contain at least one full set of System II (Ccs) genes.

## Methods

All anammox bacteria belong to the order *Brocadiales* that branches deeply into the phylum *Planctomycetes* and includes five genera (*Kuenenia, Scalindua, Brocadia, Jettenia*, and *Anammoxoglobus)*[10]. In this study draft genomes representative of four anammox genera were analyzed. *Kuenenia stuttgartiensis* [NCBI bioproject: PRJNA16685 [5]], *Scalindua profunda* [JGI: 2017108002 and 2022004002 [6]], and strain KSU-1 (representing *Jettenia* genus) [NCBI bioprojects: PRJDA163683 and PRJDB68 [7]] obtained as described elsewhere. Genomic data for *Brocadia fulgida* were obtained as described here below.

### *Brocadia fulgida* genomic data

#### Library preparation and sequencing

All kits used in this section were obtained from Life technologies (Life technologies, Carlsbad, CA, USA). Genomic DNA, isolated using a CTAB phenol/chloroform based method, was sheared for 5 minutes using the Ion Xpress™ Plus Fragment Library Kit following the manufacturer’s instructions. Further library preparation was performed using the Ion Plus Fragment Library Kit following manufacturer’s instructions. Size selection of the library was performed using an E-gel 2% agarose gel. Emulsion PCR was performed using the Onetouch 200 bp kit and sequencing was performed on an IonTorrent PGM using the Ion PGM 200 bp sequencing kit and an Ion 318 chip, resulting in 5.25 million reads with an average length of 179 bp.

#### Assembly and annotation

The obtained 5.25 million reads were quality trimmed and all reads below 200 bp were discarded. The remaining 2,22 million reads were assembled using the CLC genomics workbench (v6.5.1, CLCbio, Aarhus, Denmark) with word size 35 and bubble size 5000. *Brocadia fulgida* accounted for 91% of the assembled reads. Contigs were assigned to *Brocadia fulgida* based on coverage (>30 fold). The obtained 411 contigs were annotated using Prokka 1.7.2 (Prokka: Prokaryotic Genome Annotation System - http://vicbioinformatics.com/). After annotation, a round of manual curation was performed to correct detected frame shifts. Raw reads and assembled data are available under NCBI bioproject PRJEB4876.

### Cytochrome *c* maturation pathway

Reference protein datasets for each of the three cytochrome *c* maturation Systems (I-III) were compiled (Additional file 1), each comprising all protein and polypeptide sequences available at UNIPROT, annotated as any of the defining system-specific components (Additional files 2 and 3). A thioredoxin dataset for maturation System II was also constructed comprising UNIPROT entries for CcsX, DsbD, and CcdA. All abovementioned datasets were limited to peer-reviewed entries.

All anammox gene products were compared to the datasets using blastP (as implemented in the CLC genomics workbench, v6.5.1, CLCbio, Aarhus, Denmark) with an E-value cut off of 10^-6^. Significant hits were further analyzed by HHpred against all available HMM databases with HHBlits as the MSA generation method [11]. The web server implementation of HMMER (default settings) was also utilized [12]. Protein family matches were identified *via* Pfam (default settings) [13]. For structure- or sequence-specific feature recognition, transmembrane helical domains were predicted using the TMHMM web server [14] and potential signal peptides were annotated using SignalP 4.1 [15]. Conserved motifs and critical residues were procured from literature (Additional file 2) and probed in each gene product directly. Multiple alignments of CcsA and CcsB anammox homologs were performed using ClustalW (default settings) and phylogenetic trees were constructed based on the Maximum Likelihood algorithm utilizing the JTT matrix-based model (test of phylogeny: bootstrap method; number of replications: 1000; gaps/missing data treatment: use all sites), both as implemented in MEGA 5.0 [16]. BlastP was also utilized to search for related outgroup sequences in GenBank.

## Results & discussion

### Assignment of cytochrome *c* maturation System II in anammox bacteria

In this study, we applied comparative genomics to predict the maturation pathway of *c*-type cytochrome proteins in four anammox genera, using key protein components of maturation Systems I-III as biomarkers.

Using our approach, none of the marker genes for System I or III could be identified in the anammox draft genomes. On the contrary, our overall results evinced System II to be the dedicated *c*-type cytochrome biogenesis pathway that anammox bacteria employ.

System II, (cytochrome *c* synthesis, 'ccs’) comprises three system-specific proteins (CcsABX) together with a thiol-disulfide membrane transporter (DsbD or CcdA). According to the bacterial working model, two transmembrane proteins (CcsAB), forming a channel entry, facilitate the heme transport and the maintenance of it in a reduced state at the *p*-side of the membrane [17]. A dedicated membrane-anchored thiol-disulfide oxidoreductase (CcsX) reduces the apocytochrome *c* cysteines while reducing equivalents are transferred from a non-specific cytoplasmic thioredoxin to the thiol-disulfide membrane transporter (DsbD or CcdA) [18]. Eventually, spontaneous ligation for the thioether linkages formation takes place [17].

Following the experimental approach described above, homologs of CcsA (sometimes referred to as ResC) were successfully identified in all anammox genera; three putative CcsA proteins were found in *Kuenenia*, strain KSU-1 and *Scalindua* and two in *Brocadia* (Additional file 4). For a functional type II cytochrome *c* maturation system, complexation of CcsA and CcsB is required [17]. CcsB (sometimes called ResB) exhibits weak sequence conservation although structural homology is observed [19]. Our results further support this, since only one isoform for each *Kuenenia*, *Scalindua*, and strain KSU-1 was found by reference database search and two for *Brocadia* (Additional file 4). Nevertheless, when intra- and intergenome examination with the significant CcsB hit of *Kuenenia* as query was performed, one more CcsB isoform was retrieved for each *Kuenenia*, *Scalindua* and strain KSU-1. Results from HHpred and HMMER annotation were strikingly in agreement with those generated by blastP (compare Additional file 4 with Additional file 5). It is surprising that anammox genera contain multiple CcsB homologs; to the best of our knowledge, only one CcsB homolog has been found in any other organism to date.

Functional assignment of CcsA and CcsB is based on sequence homology [19], a minimum number of transmembrane helices and the presence of conserved motifs and essential residues (see Additional file 2). The combined results indicate that all anammox genera tested herein share a common protein pattern regarding their cytochrome *c* maturation system, all coding for two distinct CcsA-CcsB complexes (Table 1). All CcsA and CcsB homologs of *Kuenenia* and *Scalindua* were also detected in transcriptome and proteome analyses [6,20]. In detail, in the genomes of *Kuenenia*, *Brocadia*, strain KSU-1 and *Scalindua* a CcsA homolog, possessing the CcsA-specific tryptophan-rich heme-binding motif (WAXX(A/δ)WGX(F/Y)WXWDXKEXX) and 8 transmembrane helices, is found adjacent to a CcsB homolog possessing 2-4 transmembrane helices and a large soluble domain. Notably, the CcsB sequence motif (VNX_1-4_P) is found in duplicate in the canonical CcsB from strain KSU-1, whereas in *Scalindua* only a truncated CcsB motif is retrieved (VN) albeit three times. Intriguingly, the second CcsA-CcsB cytochrome *c* maturation complex encoded by all four anammox genera displays alterations from the canonical complex [19] regarding a modified CcsA heme-binding motif:

**Table 1:** CcsA and CcsB homologs identified in four anammox genera

**Anammox genus**	**Homolog**	**Gene product**	**Length (aa)**	BLAST*	HHPRED**	HMMER**	**Motif**	**His residues**	**TMHs**	**Pfam family**
***Kuenenia***	CcsA	kustd1760	283	✓	✓	✓	✓	✓	8	PF01578
	CcsB	kustd1761	629	✓	✗	✓	✓	✓	4	PF05140
	CcsA	kuste3100	257	✓	✗	✓	M	✓	8	PF01578
	CcsB	kuste3101	322	✗	✓	✓	T	✓	4	✗
**KSU-1**	CcsA	GAB62001.1	282	✓	✓	✓	✓	✓	8	PF01578
	CcsB	GAB62000.1	621	✗	✓	✓	✓	✓	4	PF05140
	CcsA	GAB64165.1	255	✓	✓	✓	M	✓	8	PF01578
	CcsB	GAB64166.1	335	✗	✓	✓	T	✓	4	✗
***Scalindua***	CcsA	scal00629c	291	✓	✓	✓	✓	✓	8	PF01578
	CcsB	scal00630c	625	✓	✓	✓	T	✓	3	PF05140
	CcsA	scal00436	258	✓	✓	✓	M	✓	8	PF01578
	CcsB	scal00437	322	✗	✓	✓	✗	✓	4	✗
***Brocadia***	CcsA	BFUL_01704	281	✓	✓	✓	✓	✓	8	PF01578
	CcsB	BFUL_01703	499	✓	✓	✓	✓	✓	2	PF05140
	CcsA	BFUL_02788	255	✓	✓	✓	M	✓	8	PF01578
	CcsB	BFUL_02789	319	✓	✓	✓	T	✓	4	PF05140

Legend: Initial blastP search of whole anammox genomes against a reference database (for details see Additional file 3), comprising UNIPROT entries for CcsA and CcsB, together with intra- and intergenome searches with the significant hits from *Kuenenia* as queries were performed (Additional file 4). Retrieved results were further analyzed with HHpred and HMMER (Additional file 5), transmembrane helices were predicted with TMHMM, protein family matches were identified via Pfam, and conserved motifs together with critical residues were identified manually. Regarding the motif search, symbol (✓) denotes identification of the canonical motif as known from the literature (CcsA: WAXX(A/δ)WGX(F/Y)WXWDXKEXX and CcsB: VNX_1-4_P), letter (M) denotes presence of the CcsA modified heme-binding motif as found in the anammox genera tested (WGXXAWGXYFLWDAK(V/L)(V/L)W), and letter (T) denotes presence of the truncated CcsB motif (VN). TMHs: transmembrane helices; (*): E-value cut off set at 10^-6^; (**): E-value cut off set at 10^-3^; (✓): significant annotation and/or identification; (✗): absence of significant hits and/or protein matches.

*Published:* W A X X (A/S) W G X (F/Y) W X W D X K E X X

*Modified:* W G X X A W G X Y F L W D A K (V/L) (V/L) W

**Figure 2:**
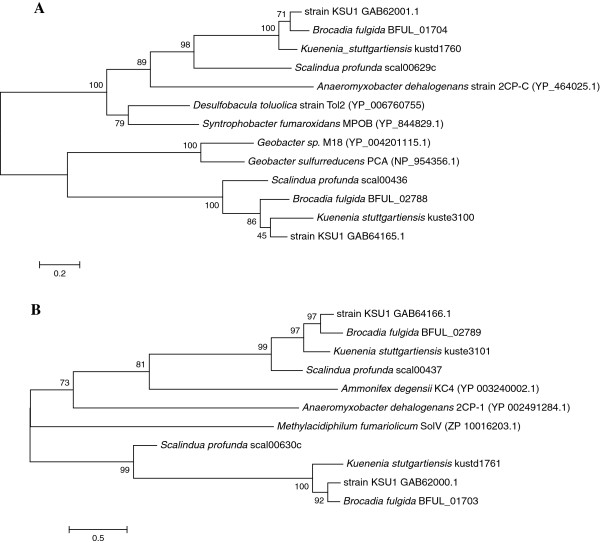
**Unrooted phylogenetic trees, constructed based on the Maximum Likelihood algorithm, indicating the relationships of CcsA (A) and CcsB (B) homologs of four anammox genera.** Anammox CcsA and CcsB homologs were used as queries for blastP annotation and five (for CcsA) or three (for CcsB) significant hits were included in the construction of the tree. NCBI accession numbers of reference sequences are shown in parentheses. The evolutionary history was inferred by using the Maximum Likelihood method based on the JTT matrix-based model [21]. The tree with the highest log likelihood (-6044.3478 for CcsA; -11148.2432 for CcsB) is shown. The percentage of trees in which the associated taxa clustered together is shown next to the branches. Initial tree(s) for the heuristic search were obtained by applying the Neighbor-Joining method to a matrix of pairwise distances estimated using a JTT model. The tree is drawn to scale, with branch lengths measured in the number of substitutions per site. All ambiguous positions were removed for each sequence pair. There were a total of 401 and 685 positions in the final dataset for CcsA and CcsB, respectively. Evolutionary analyses were conducted in MEGA 5.0 [16].

In the latter, the observed amino acid substitutions may suggest a structurally different heme-binding configuration and/or implications for protein functionality. Nonetheless, the identified CcsA and CcsB homologs are coded adjacent to each other in all anammox genomes. Phylogenetic relationships among the anammox CcsA and CcsB homologs are illustrated in Figure 2A and 2B, respectively.

Along with a functional CcsA-CcsB complex, cytochrome *c* maturation System II further requires an efficient thiol-reduction pathway through which reducing equivalents are shuttled across the energy-transducing membrane towards the *p*-side, and are subsequently used for reduction of apocytochrome *c* cysteines [18]. In all four anammox species we studied, DsbD, a thiol-disulfide membrane transporter involved in the aforementioned pathway, is annotated successfully and with high confidence by a similar comparative methodology adopted for CcsA and CcsB (Table 2 and Additional file 6). In detail, two DsbD homologs are identified in *Kuenenia* whereas a single copy is retrieved for strain KSU-1 and *Brocadia*. All DsbD homologs share similar structural features, including 8-11 transmembrane helices and conserved cysteine residues [22]. *Scalindua* contains a homolog of CcdA, related to but shorter than DsbD, possessing only 6 transmembrane helices along with two cysteine residues [23]. DsbD is a housekeeping thiol-disulphide electron shuttle [24] and as such it is not an indispensable cytochrome *c* maturation System II component. In contrast, CcsX (sometimes called ResA) that fulfils the essential role of apocytochrome *c* reduction in this disulfide bond cascade is a dedicated membrane-anchored thiol-disulfide oxidoreductase of maturation System II. Apart from the conserved thioredoxin cytochrome *c* recognition motif (CXXC), CcsX also possesses additional cysteine residues and a single transmembrane helix through which it is anchored to the membrane. Our comparative computational approach identified multiple potential CcsX homologs for each anammox genus. Particularly, two CcsX-like homologs for *Brocadia*, three for *Kuenenia* and six for each, *Scalindua* and strain KSU-1, were identified with high confidence (Additional file 6). However, homologs possessing no signal peptide sequences were ruled out from our final collective table (Table 2). Although distinction between the dedicated CcsX proteins and other thioredoxins that might possess similar features cannot be made, the presence of that many CcsX-like homologs suffices for a complete *c*-type cytochrome maturation System II.

**Table 2:** CcsX and DsbD homologs identified in four anammox genera

**Homolog**	**Anammox genus**	**Gene product**	**Length (aa)**	BLAST*	HHPRED**	HMMER**	**Motif**	**Additional Cys residues**	**TMHs**	**Signal peptide**
**CcsX**	***Kuenenia***	kuste0860	161	✓	✓	✓	CX_2_C	1	✓	✗
		kuste0967	166	✓	✓	✓	CX_2_C	1	✓	✗
		kuste3827	164	✓	✓	✓	CX_2_C	3	✓	✓
	***Scalindua***	scal02124	172	✓	✓	✓	CX_2_C	0	✓	✓
		scal00014c	173	✓	✓	✓	CX_2_C	3	✓	✓
		scal02421c	255	✓	✓	✓	CX_2_C	1	✓	✓
		scal02845	125	✓	✓	✓	CX_2_C	0	✗	✗
		scal00012c	185	✓	✗	✓	CX_2_C	1	✗	✓
		scal04176	164	✓	✓	✓	CX_4_C	1	✗	✗
	**KSU-1**	GAB64172.1	312	✓	✓	✓	CX_2_C	1	✓	✗
		GAB61322.1	165	✓	✓	✓	CX_2_C	2	✓	✓
		GAB62714.1	162	✓	✓	✓	CX_2_C	1	✓	✓
		GAB64222.1	163	✓	✓	✓	CX_2_C	1	✓	✓
		GAB64221.1	163	✓	✓	✓	CX_2_C	0	✓	✓
		GAB62039.1	669	✓	✓	✓	CX_2_C	8	✓	✗
	***Brocadia***	BFUL_03119	163	✓	✓	✓	CX_2_C	0	✓	✓
		BFUL_00886	173	✓	✓	✓	CX_2_C	2	✗	✓
**DsbD**	***Kuenenia***	kuste2732	601	✓	✓	✓	NA	7	8	NA
		kustc0946	608	✓	✓	✓	NA	8	9	NA
	**KSU-1**	GAB61320.1	610	✓	✓	✓	NA	5	11	NA
	***Brocadia***	BFUL_00929	610	✓	✓	✓	NA	5	9	NA
**CcdA**	***Scalindua***	scal01537	234	✓	✓	✓	NA	2	6	NA

Legend: Initial blastP search of whole anammox genomes against a reference database, comprising UNIPROT entries for CcsX and DsbD was performed. Retrieved results were further analyzed with HHpred and HMMER (Additional file 6), transmembrane helices were predicted with TMHMM, potential signal peptides were annotated using SignalP 4.1, and conserved motifs together with critical residues were identified manually. TMHs: transmembrane helices; (*): E-value cut off set at 10^-6^; (**): E-value cut off set at 10^-3^; (✓): significant annotation and/or identification; (✗): absence of significant hits and/or transmembrane helix and/or signal peptides; (NA): not applicable.

Overall, these results indicate that the assembly of cytochrome *c* holoforms is achieved by the maturation System II in all anammox bacteria tested herein. All genera code for at least one CcsA-CcsB complex, one DsbD (or CcdA), and one CcsX homolog, all being essential components of a functional cytochrome *c* maturation System II.

### Working model

Having analyzed the cytochrome *c* maturation system in anammox bacteria, it would be stimulating to comprehend how such machinery is localized within the intricate anammox cell plan. A hypothetical cellular pathway for cytochrome *c* biogenesis is illustrated in Figure 1B. According to our view, the CcsA-CcsB complex, forming the heme channel entry, must be tethered within the anammoxosome membrane. Heme is, thus, translocated into the anammoxosome, with the latter representing the *p*-side of the anammox cell [3]. This translocation is mediated by selective CcsA heme-binding motifs (as specified in Table 1). Concurrently, housekeeping riboplasmic thioredoxins provide DsbD with the necessary reductants that are shuttled towards the dedicated CcsX thiol-disulfide oxidoreductase. Both DsbD and CcsX possess transmembrane helices spanning the anammoxosome membrane, with the CcsX globular domain facing the inside of the anammoxosome, where apocytochrome *c* cysteine reduction occurs. Eventually, spontaneous formation of the thioether linkages between the apoprotein and its cofactor takes place, leading to functional cytochrome *c* holoforms inside the anammoxosome [4].

## Conclusions

These findings suggest that anammox bacteria possess at least one complete machinery for type II cytochrome *c* biogenesis [19], adapting it to their complicated cell plan; the anammoxosome membrane is proposed to be the main site of cytochrome *c* maturation. Our results provide a working model that will be used to guide experimental studies, including protein purification and immunogold electron microscopy, in elucidating both the localization and the function of cytochrome *c* maturation System II in anammox bacteria.

### Supporting data

The data sets supporting the results of this article are included within the article and its additional files.

## Competing interests

The authors declare that they have no competing interests.

## Authors’ contribution

CF, JWAA and MSMJ conceived of the study. DRS sequenced and analyzed the genomic data of *Brocadia.* CF built the datasets and ran homologue searches. DRS, JR, JTMK, and HJMOC assisted in bioinformatics analysis and data interpretation. CF, JWAA, and MSMJ wrote the manuscript with input from all co-authors. All authors read and approved the final manuscript.

## Supplementary Material

Additional file 1 **Reference protein datasets for cytochrome *c *** **maturation Systems (I-III) and thioredoxin dataset for System II.** Click here for file

Additional file 2 **Cytochrome *c *maturation System biomarkers.** For each cytochrome *c* maturation System (I-III), essential protein components that can be used as suitable biomarkers for annotation purposes were selected (for details see Additional file 3) and their defining characteristics are listed herein. Click here for file

Additional file 3 **Selection criteria for cytochrome *c *** **maturation System biomarkers.** Click here for file

Additional file 4 **CcsA and CcsB homologs identified in four anammox genera using blastP.** Homology identification was performed with blastP as implemented in CLC genomics workbench (v6.5.1, CLCbio, Aarhus, Denmark). Whole anammox genomes are used as queries against a reference database that comprises all reviewed entries for CcsA and CcsB available at UNIPROT. An E-value of 10^-6^ was set as cut off to prevent ambiguity. Click here for file

Additional file 5 **CcsA and CcsB homologs identified in four anammox genera using HHpred and HMMER.** Homology identification was performed with blastP as implemented in CLC genomics workbench (v6.5.1, CLCbio, Aarhus, Denmark). Whole anammox genomes are used as queries against a reference database that comprises all reviewed entries for CcsA and CcsB available at UNIPROT. Intra- and intergenome searches with the significant hits from *Kuenenia* as queries were also performed (Additional file 4). Retrieved results were further analyzed with HHpred and HMMER. An E-value of 10^-3^ was set as cut off to prevent ambiguity. Click here for file

Additional file 6 **CcsX and DsbD homologs identified in four anammox genera using blastP, HHpred and HMMER.** Homology identification was performed with blastP as implemented in CLC genomics workbench (v6.5.1, CLCbio, Aarhus, Denmark). Whole anammox genomes are used as queries against a reference database that comprises all reviewed entries for CcsX and DsbD available at UNIPROT. Retrieved results were further analyzed with HHpred and HMMER. (*): E-value cut off set at 10^-6^; (**): E-value cut off set at 10^-3^. Click here for file
