# Supplementary material for: Identification of the type II cytochrome c maturation pathway in anammox bacteria by comparative genomics
Source: BMC Microbiol. 2013 Nov 23;13:265. doi: 10.1186/1471-2180-13-265 (PMC4222556; doi:10.1186/1471-2180-13-265)
Supplement: Additional file 3 — Selection criteria for cytochrome c maturation System biomarkers. [file 1471-2180-13-265-S3.pdf]

System I

As thoroughly explained elsewhere [1], the cytochrome *c* maturation System I apparatus comprises a variety of proteins which are finely orchestrated to perform the seemingly simple task of cytochrome *c* assembly. Nonetheless, not all protein components can be used as reliable computation biomarkers towards the identification of this maturation system. CcmA and CcmB both belong to the ATP-Binding Cassette (ABC) transporters superfamily, which exhibits high sequence conservation within its members. Most notably, their ATP-binding domains include two short motifs (Walker motifs) associated with many nucleotide-binding proteins [2] and, thus any annotation attempt would result in several non-specific hits. CcmG is a specific thiol-oxidoreductase but, as such, it adopts a trx-like fold which classifies it as a thioredoxin [3] and makes it completely inappropriate as a signature gene, considering the ubiquitous nature of these proteins in all living cells. CcmH from *E.coli* is, in most organisms, divided into two proteins, CcmH and CcmI, with the former being a thiol-oxidoreductase albeit with an unusual structural fold [4]. Although inability to detect CcmH during an annotation project does not necessarily exclude System I from being the dedicated cytochrome *c* maturation pathway of the organism [5], identification of CcmH can offer supportive indication towards that conclusion. CcmI, on the other hand, contains TPR repeat domains and, therefore cannot be used as a biomarker [1]. Detection of the system-specific heme-handling genes in the genome of an organism can offer strong evidence for the presence of cytochrome *c* maturation System I. CcmE, a heme chaperone, represents a novel class of covalently heme-bound proteins [6] and contains a highly conserved motif (C/H)XXXY). CcmC and CcmF, like CcsA in System II, contain a typical tryptophan-rich WWD domain, flanked by conserved histidine residues [7]. CcmD, at last, is not the most suitable biomarker due to its small size and poor sequence conservation, although it can

further validate the possible presence of CcmC, since it is always downstream of the latter and adopts a well preserved domain structure [8].

System II

In silico identification of cytochrome *c* maturation System II proceeds in a heuristic manner that is based on two indispensable Ccs proteins (CcsA and CcsB) that form a tight complex and perform most of the tasks required for cytochrome *c* holoform assembly. Although in some organisms a fused CcsBA is the dedicated cytochrome *c* synthetase [9], in most cases two separate gene products are identified. CcsA exhibits high sequence conservation and, in resemblance with CcmC and CcmF from System I, contains a WWD domain and flanking histidines [1]. CcsB, which usually proceeds or follows CcsA in the genome, is poorly conserved at the sequence level, even though its conserved secondary structure could be of help. The fused CcsBA also exhibits highly conserved features [10].

System III

Cytochrome *c* maturation System III has so far been detected only in eukaryotes and seems to have the simplest protein composition of all. The prototypical cytochrome *c* heme lyase (CCHL) together with a related type of it (CC_1HL; responsible for cytochrome c_1 maturation in some organisms) [11] are the only defining components of this system described up to now [12]. Together with these, a dedicated flavoprotein (Cyc2p), suggested to be involved in the redox pathway of System III cytochrome *c* assembly in fungi [13] and the human cytochrome *c* heme lyase (HCCS) were also included in our dataset.

References

1. Kranz RG, Richard-Fogal C, Taylor JS, Frawley ER: **Cytochrome *c* biogenesis: mechanisms for covalent modifications and trafficking of heme and for heme-iron redox control.** *Microbiol Mol Biol Rev* 2009, **73:**510-528.
2. Higgins CF: **ABC transporters: from microorganisms to man.** *Annual review of cell biology* 1992, **8:**67-113.
3. Di Matteo A, Calosci N, Gianni S, Jemth P, Brunori M, Travaglini-Allocatelli C: **Structural and functional characterization of CcmG from *Pseudomonas aeruginosa*, a key component of the bacterial cytochrome c maturation apparatus.** *Proteins: Structure, Function, and Bioinformatics* 2010, **78:**2213-2221.
4. Di Matteo A, Gianni S, Schininà ME, Giorgi A, Altieri F, Calosci N, Brunori M, Travaglini-Allocatelli C: **A Strategic Protein in Cytochrome *c* Maturation.** *Journal of Biological Chemistry* 2007, **282:**27012-27019.
5. Allen JWA, Harvat EM, Stevens JM, Ferguson SJ: **A variant System I for cytochrome *c* biogenesis in archaea and some bacteria has a novel CcmE and no CcmH.** *FEBS letters* 2006, **580:**4827-4834.
6. Thony-Meyer L: **A heme chaperone for cytochrome *c* biosynthesis.** *Biochemistry* 2003, **42:**13099-13105.
7. Lee JH, Harvat EM, Stevens JM, Ferguson SJ, Saier MH, Jr.: **Evolutionary origins of members of a superfamily of integral membrane cytochrome *c* biogenesis proteins.** *Biochim Biophys Acta* 2007, **1768:**2164-2181.
8. Ahuja U, Thöny-Meyer L: **CcmD is involved in complex formation between CcmC and the heme chaperone CcmE during cytochrome *c* maturation.** *Journal of Biological Chemistry* 2005, **280:**236-243.
9. Simon J, Kern M, Hermann B, Einsle O, Butt JN: **Physiological function and catalytic versatility of bacterial multihaem cytochromes *c* involved in nitrogen and sulfur cycling.** *Biochem Soc Trans* 2011, **39:**1864-1870.
10. Beckett CS, Loughman JA, Karberg KA, Donato GM, Goldman WE, Kranz RG: **Four genes are required for the System II cytochrome *c* biogenesis pathway in *Bordetella pertussis*, a unique bacterial model.** *Molecular microbiology* 2000, **38:**465-481.
11. Zollner A, Rodel G, Haid A: **Molecular cloning and characterization of the *Saccharomyces cerevisiae* CYT2 gene encoding cytochrome c_1 heme lyase.** *Eur J Biochem* 1992, **207:**1093-1100.
12. Hamel P, Corvest V, Giege P, Bonnard G: **Biochemical requirements for the maturation of mitochondrial *c*-type cytochromes.** *Biochim Biophys Acta* 2009, **1793:**125-138.
13. Bernard DG, Quevillon-Cheruel S, Merchant S, Guiard B, Hamel PP: **Cyc2p, a membrane-bound flavoprotein involved in the maturation of mitochondrial *c*-type cytochromes.** *J Biol Chem* 2005, **280:**39852-39859.
